# SRS143 a semi-synthetic analogue of andrographolide against house dust mite induced allergic asthma

**DOI:** 10.1007/s00210-025-04589-8

**Published:** 2025-10-25

**Authors:** Chee Yi Tan, Okwuofu Emmanuel Oshiogwe, Anoosha Saud, Hui Suan Ng, Sreenivasa Rao Sagineedu, Johnson Stanslas, Jonathan Chee Woei Lim

**Affiliations:** 1 Department of Medicine, Faculty of Medicine and Health Sciences, Universiti Putra Malaysia, Serdang 43400, Selangor, Malaysia; 2 Department of Chemical Engineering and Materials Science, Yuan Ze University, Chungli, Taoyuan 320 Taiwan; 3 Department of Chemisty, IMU University, 126, Jln Jalil Perkasa 19, Bukit Jalil, 57000 Kuala Lumpur, Federal Territory of Kuala Lumpur Malaysia; 4 Institute for Research, Development and Innovation (IRDI), IMU University, 126, Jln Jalil Perkasa 19, Bukit Jalil, 57000 Kuala Lumpur, Federal Territory of Kuala Lumpur Malaysia; 5 Department of Pharmacology, Faculty of Medicine, MAHSA University, 42610 Jenjarom, Selangor Malaysia

**Keywords:** Andrographolide, Andrographolide analogue, Allergic asthma, Mast cell degranulation, Anti-histamine

## Abstract

Allergic asthma is one of the common forms of asthma. Upon exposure to allergens, mast cells actively release histamine into the airway, forcing the smooth muscles in the airway to undergo contraction, hence asthmatic attack. The therapeutics of asthma is commonly based on the use of steroids and topical bronchodilators. Steroid-resistant asthma has been on the rise and can be further exacerbated by any respiratory infection. Treatment requires a high dose of steroids to produce efficacy and persistent use of high-dose steroids will result in debilitating side effects. There is a need for better and safer alternatives with a different therapeutic mechanism than steroids to complement existing therapies. Andrographolide (AGP) and its analogues have been shown to display potent anti-inflammation effects in many various asthma models. Despite having potent anti-asthma activity, AGP and analogues did not display any anti-histamine activity against histamine release assay. Surprisingly SRS143, an AGP analogue displayed improved potency in both in vitro and in vivo allergic models. Inhibition of histamine release by antigen-activated mast cells was observed without causing any toxicity. Similar observations were also seen in our in vivo model whereby asthmatic BALB/c female mice were induced by sensitising and challenging them with house-dust mite (HDM). Animals administered with SRS143 intraperitoneally had shown potent inhibition on asthmatic responses. SRS143 treated asthmatic mice displayed significant reduction of pulmonary eosinophilia, airway hyper-responsiveness, and mucus hypersecretion. This potent analogue warrants a potential treatment for allergic asthma.

**Supplementary Information:**

The online version contains supplementary material available at 10.1007/s00210-025-04589-8.

## Introduction

Globally asthma is estimated to affect over 262 million people of all ages with 461,000 deaths annually (Asher et al. 2020a). Despite advances in asthma management, the control of the disease is reported to be poor (Gold et al. 2014), with variation according to regions. High-income countries such as Australia, United Kingdom, and United States have reported rates of about 50% of patients with asthma as being well-controlled (Dharmage et al. 2019; García-Marcos et al. 2023). In the Asia–Pacific region, only 7.6% of patients with asthma aged below 12 years were well-controlled (Gold et al. 2014).

The prevalence of asthma is high among children, with urban areas showing a disproportionately higher burden (Dinglasan et al. 2022). Recent estimates indicate global prevalence rates of 11% among children aged 6–7 years, 9.1% in those aged 13–14 years, and 8.6% among adults (Dinglasan et al. 2022; García-Marcos et al. 2022; Asher et al. 2020b). Only a small percentage of the 6% of patients with asthma aged below 12 years had well-controlled symptoms (Gold et al. 2014). Allergic asthma (AA) is the most common type of asthma, characterized by airway inflammation, mucus hypersecretion, and airway hyper-responsiveness, induced and triggered by an aeroallergen (Akar-Ghibril et al. 2020). The onset of AA occurs during childhood and is often associated with comorbidities such as atopic dermatitis and allergic rhinitis. Management of AA relies heavily on steroids and topical bronchodilators. Considering the long-term use of oral/systemic steroids, detrimental, and significant adverse effects are always reported (Poetker and Reh 2010; Yasir et al. 2024). Depending on cellular and molecular characterisation of the inflammatory cascade, asthma may either be allergic or non-allergic endotype. AA is usually eosinophilic, whereas non-allergic endotypes, such as aspirin, infection and exercise induced asthma may present with neutrophilic or paucigranulocytic phenotype (Copyright © 2024, StatPearls Publishing LLC. 2024). Mast cells are notorious for their detrimental impact on a number of pathological conditions, including asthma and other allergic conditions (Sze et al. 2020). Besides basophils and dendritic cells, mast cells highly express high-affinity IgE receptor FcγR1 which binds to IgE antibodies produced by type 2 cytokine-induced B cells (Bax et al. 2012). When mast cells are activated by antigen-mediated crosslinking of IgE bound to their high-affinity cell surface receptors FcγR1, they respond by degranulation, whereby potent inflammatory molecules, including histamine, inflammatory lipid mediators, and chemokines are released. This is followed by airway hypersensitivity. Mast cells and the extent of its degranulation have been found within the airway smooth muscles cell layer, indicating a strong correlation with asthma while uncontrolled asthma seems associated with infiltration of mast cells into the lung parenchyma (Banafea et al. 2022).

**Fig. 1:**
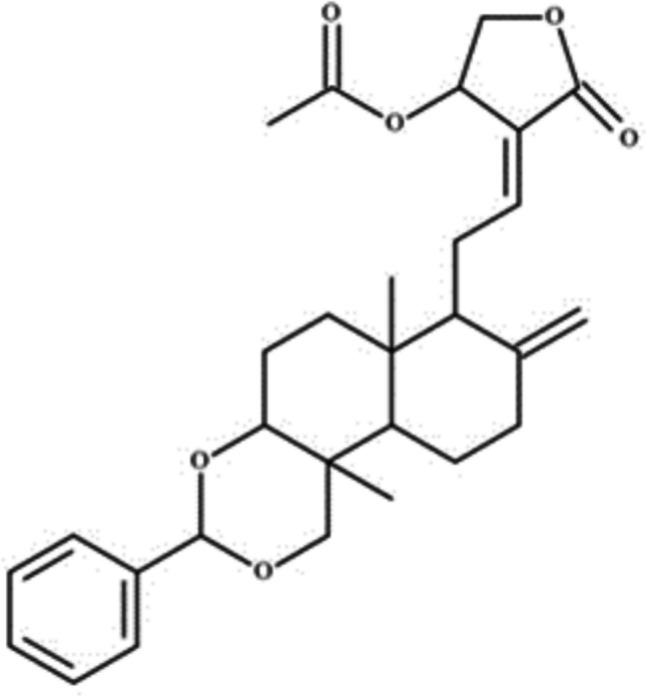
Chemical structure of 3,19-Benzylidene-14-acetylandrographolide (SRS143)

**Fig. 2:**
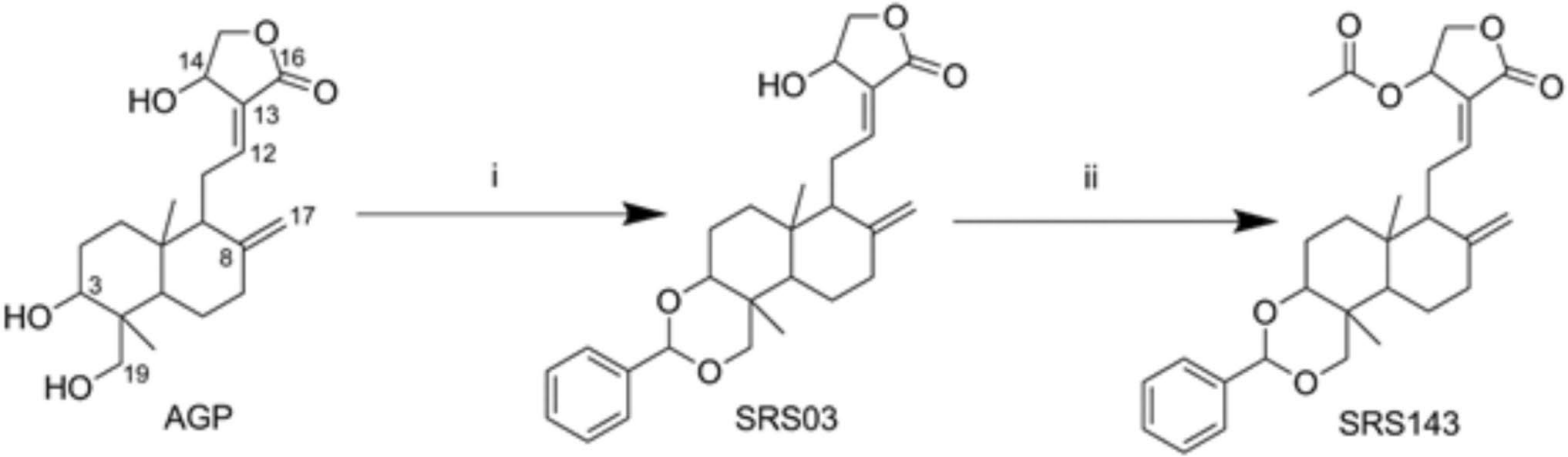
Synthesis scheme of SRS143 using AGP as template. The reagents and conditions involved in (i) and (ii): (i) pyridinium p-toluenesulfonate, benzaldehyde, benzene–DMSO (9:1), reflux, 4 h (ii) acetic anhydride, DMAP, DCM, 0 °C, 1 h

*Andrographis paniculata* (AP) plant and its major active compounds such andrographolide (AGP) and 14-deoxy-11,12-didehydroandrographolide (DDAG) have been shown to have various therapeutic activities (Pejler 2019). Most of the studies involving histamine release focus only on AP, not AGP and its analogues. A study conducted by Karpakavalli et al. showed that the hydroalcoholic extract of AP leaves possessed antihistamine effect in a guinea pig ileum experimental model. Animals treated with the AP extract displayed reduced histamine (Lim et al. 2012) concentration compared to the normal animals. In another studies, AP plant extract and its major compounds in an in vitro calcium ionophore were exposed to A23187 mediated mast cell degranulation model, in which only 7-O-methylwogonin was reported to have antihistamine activity (Karpakavalli, et al. 2010). On the contrary, alcohol and ethyl acetate extract fractions of AP AGP, DDAG and neoandrographolide were found to increase P815 cell degranulation and histamine level (Chandrasekaran et al. 2011). To date, there have been no reports on the inhibition of histamine release by AGP, DDAG, or their derivatives in calcium ionophore- or IgE-mediated mast cell models. AGP has been reported to have extremely varied pharmacological activities including antiinflammatory, immunostimulatory, antitumour,, cytotoxicity and cardioprotectivity, and antiasthma activities (Hu et al. 2015; Gupta et al. 2020; Jiaqi et al. 2023; Phetruen et al. 2023) but not antihistamine activity. Our lab investigation on a series of andrographolide analogues for the treatment of allergic asthma revealed one of the andrographolide analogues was found to potently inhibit histamine release in both calcium ionophore A23187 activated and IgE-FceRI receptor stimulated mast cell models. The chemical structure of 3,19-Benzylidene-14-acetylandrographolide, an andrographolide analogue is shown in Fig. 1, while Fig. 2 illustrates the pathway for the synthesis.

## Methodology

### Synthesis of SRS143

Synthesis of SRS143 (3,19-Benzylidene-14-acetylandrographolide) using AGP as starting material. Briefly, a solution of AGP (1 mmol) in benzene-DMSO (50 mL, 9:1 v/v) was refluxed with aromatic aldehyde (2–4 mmol eq) and catalytic pyridinium p-toluenesulphonate for 1–4 h. After TLC-confirmed completion, the mixture was cooled, quenched with triethylamine, diluted with benzene, washed (water, brine), dried (Na₂SO₄), filtered, concentrated, and purified by centrifugal chromatography on silica gel using DCM–MeOH (98:2). Benzylideneandrographolide (1 mmol) reacted with acetic anhydride, DMAP, and triethylamine in DCM at 0 °C, followed by TLC-confirmed workup, extraction, and silica gel chromatography.

### Cell viability assay

RBL-2H3 cells were maintained in Minimum Essential Media (MEM, ThermoFischer, Grand Island, NY), supplemented with 15% heat-inactivated FBS, 100 U/mL of penicillin and 100 µg/mL of streptomycin. The cytotoxicity effect of the SRS143 was carried out using the rapid colorimetric assay that measures the activity of mitochondrial dehydrogenase in living cells, which convert the pale yellow soluble tetrazolium salt MTT into a insoluble purple end product formazan.

### β-hexosaminidase release assay

The inhibitory activity of SRS143 and quercetin against the release of β-hexosaminidase from RBL-2H3 cells was evaluated according to the method reported previously (Lin et al. 2020). Briefly, the cells were cultured in a 24-well cell culture plate (9 × 10^5^/well, Becton–Dickinson Co., Franklin Lakes, NJ) with concentration (5 mg/ml) of mouse monoclonal anti-dinitrophenyl (DNP) IgE. After washing with PBS, SRS143 at various concentrations (0, 0.1, 1 and 10 μM)) or quercetin (30 μM) was added. After 60 min of incubation, DNP-labeled bovine serum albumin (DNP-labeled BSA, 50 ng/ml final concentration) was added, followed by 45 min of incubation. The supernatant (50 μl) was mixed with 100 μl of a 0.1 M citrate buffer (pH 4.5) containing 1 mM p-nitrophenyl-2-acetoamide-ß-D-glucopyranoside and the mixture was incubated in a 96-well plate at 37˚C for 60 min. For the calcium ionophore stimulation method, CaI A23187 was added at 5 µM to induce degranulation. Each well was replaced with 0.4 mL of MEM media and 100 µL of MTT solution to evaluate cell viability using MTT assay. Supernatants from CaI A23187-induced degranulated cells were collected, centrifuged, and reacted with PNAG in citrate buffer at 37℃ for 1 h. The reaction was stopped, and absorbance at 405 nm was measured using a microplate reader (Versamax).to assess beta-hexosaminidase activity, indicating enzyme subproduct levels. The results are expressed as percentage release of β-hexosaminidase activity amount by antigen (DNP-labeled BSA) alone.

### Immunoblotting

Nuclear proteins of RBL-2H3 cells (10 µg per lane) were separated by 10% SDS-PAGE and immunoblots were developed as described (Matsuda et al. 1994). Immunoblots were probed with anti-AKT and anti-β-actin antibodies (all from Cell Signalling Technology, Beverly, MA).

### Animals

BALB/c mice of 6–8 weeks of age were housed in the animal experimentation unit, Faculty of Medicine and Health Sciences, Universiti Putra Malaysia (UPM). Mice were kept in individually vented cages (1820 cm^3^, not more than 6 mice per cage) with a bed of poplar shaving and free access to clean water and food. Mice were grouped into 8 groups of 6 mice per group – Group 1: Normal control, Group 2: HDM, Group 3: Vehicle (6% DMSO), Group 4: SRS143 (0.3 mg/kg), Group 5: SRS143 (1 mg/kg), Group 6: SRS143 (3 mg/kg), Group 7: dexamethasone (1 mg/kg), Group 8: andrographolide (1 mg/kg). Throughout the study, both experimental and control mice were kept under appropriate conditions (22 ± 2 ^0^C temperature, and 55 ± 10% relative humidity). The mice were maintained under a 12-h light-darkness cycle.

### House dust mite allergic asthma mouse model

**Fig. 3:**
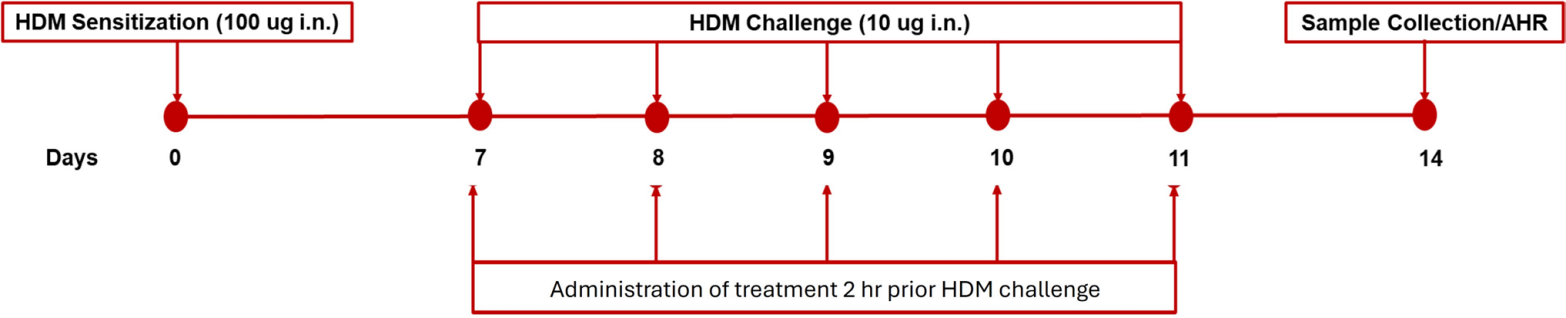
HDM-asthma induction protocol. Animals were intranasally (*i.n.)* sensitized using 100 μg of house dust mite (HDM) on day 0, this was followed by daily HDM intranasal challenge (10 μg) on day 7 −11. The treatments (6% DMSO, SRS143 (0.3 mg/kg, 1 mg/kg, 3 mg/kg), dexamethasone (1 mg/kg), andrographolide (1 mg/kg)) were administered *i.p* 2 h before HDM challenge according to the test group. Therefore, the treatments were administered for five consecutive days, starting from day 7 to day 11 of the asthma induction protocol. BAL fluid samples were collected for analysis on day 14, while animals that were scheduled for airway hyperresponsiveness (AHR) test proceeded with their respective test

Allergic asthma was induced using house dust mite (Dermatophagoides pteronyssinus, Lot Number: 253983, Greer Laboratories, Lenoir, USA). The HDM was dissolved and prepared to a concentration of 5000 µg/mL in PBS, and then it was aliquoted and frozen (−20 °C) for further use. A 14-day HDM model was used to induce asthma in Balb/c mice (Lim et al. 2016). The animals were sub-anesthetized using a cocktail of ketamine (50 mg/kg) and xylazine (3.3 mg/kg) for the intranasal HDM sensitization and challenged protocol. Briefly, the induction involved initial intranasal administration of 100 μg HDM extract (10 μL per nostril) on day 0, and subsequent administration of 10 μg of HDM (10 μL per nostril) on day 7—11. Mice were treated with SRS143 at doses of 0.3, 1, or 3 mg/kg, or with dexamethasone (1 mg/kg) or andrographolide (1 mg/kg). All treatments were administered at a constant volume of 10 mL/kg. Bronchoalveolar lavage (BAL) fluid collection was conducted on day 14. The allergic asthma induction protocol is summarized in Fig. 3.

### Bronchoalveolar lavage fluid analysis

BAL fluid total and differential cell counts were determined as described (Matsuda et al. 1994).

### Histologic and immunohistochemistry analysis

Isolated lungs were fixed in 10% neutral formalin and processed. Lung Sects. (5 µm) were stained with H&E for inflammatory cell infiltration and Periodic Acid Schiff Fluorescence stain (PAS) for mucus production. Quantitative analysis under a light microscope was then performed. To avoid bias, two independent researchers, blinded to the treatment groups, performed the scoring.

### Measurements of airway hyperresponsiveness

The animals were initially anesthetized using a mixture of ketamine (100 mg/kg), xylazine (10 mg/kg) and acepromazine (10 mg/kg). Tracheotomy was performed and mice were ventilated by a pump ventilator. The animals were placed inside a semi-invasive plethysmograph (BUXCO) to measure airway resistance (Ri) and dynamic compliance (Cdyn) in response to increasing doses of methacholine given intranasally. First, the plethysmograph is calibrated, and the mice are placed in the chamber to acclimatize for 5–10 min. Baseline recordings are then obtained, followed by PBS administration. This is then followed by nebulizing graded doses of methacholine challenges at 2, 4, 8, 16, and 32 mg/kg, each administered over approximately 3 min.

### Statistical analysis

All data are presented as mean ± SD. Comparisons was done between the disease model and treatment groups using one-way analysis of variance (ANOVA) followed by Bonferroni’s post-hoc test. SPSS 20 for Windows (SPSS Inc, Chicago, IL, USA) and Graphpad Prism 9.0.2 was used to determine significant differences among groups and graphing. For the non-parametric data, Kruskal Wallis test was done followed by Dunn’s post hoc. The critical level for statistical significance was set at p < 0.05.

## Results

### Inhibition of mast cell degranulation by AGP and derivatives

**Fig. 4:**
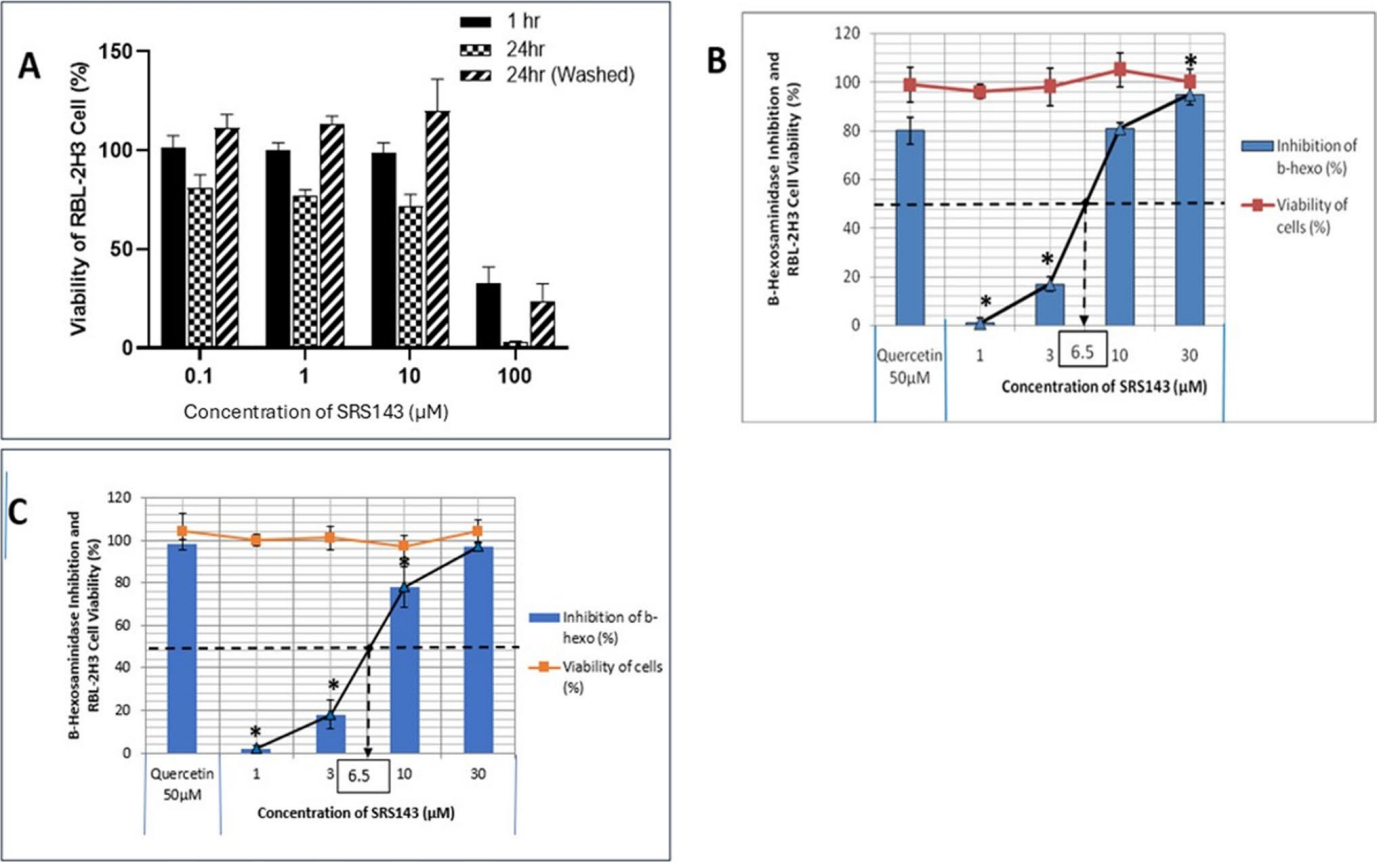
(**A**) Cytotoxicity of SRS143 on RBL-2H3 Cells at 1, 24 and 24 h (cells washed with PBS after 1 h treatment to remove compound were replaced with culture media and left to incubate till 24 h). B and C shows the effect of SRS143 on cell viability and β-hexosaminidase activity in RBL-2H3 cells (**B**) calcium ionophore A23187 (**C**) IgE-FceRI stimulation methods at 1 h. Percentage inhibitions are expressed as mean values ± S.D. of three independent experiments. Values are significantly (*p < 0.001) different when compared with Quercetin 50 µM

Mast cell degranulation is the key process in allergic asthma. To induce mast cell degranulation process in RBL-2H3 cells, the cells were sensitized and challenged with DNP-labelled specific antigen IgE. Alternatively, the mast cells were induced with calcium ionophore A23187 to trigger direct degranulation independent of activation of IgE. The release was assessed with β-hexosaminidase assay whereby the amount β-hexosaminidase enzyme released correlates to histamine release by RBL-2H3 cells. In the preliminary screening, single low dose of 10 µM of AGP and its derivatives were applied to the calcium ionophore activated cells for a short duration of 1 h. Quercetin as positive control was also applied at a higher dose of 50 µM. For cell viability assay, the cells were washed and replenished with fresh medium supplemented with 15% FBS after 1 h of treatment. The cell viability was measured at 24 h. Among the analogues, only SRS133 and SRS143 had the inhibition of 53.8% and 85.2% respectively, exceeding 50 percent without causing any toxicity. The remaining analogues (SRS21, SRS30, SRS50, SRS77 and SRS84), AGP and DDAG had a low percentage inhibition level (Table 1). Majority of the analogues except SRS21, SRS30, SRS50 and SRS83 were found to be non-toxic. Given close to eightfold improved potency in inhibition of mast degranulation (Fig. 4A, B and C). We further measured the efficacy of SRS143 in a dose response study involving both efficacy and toxicity.

**Table 1:** Inhibition of β-Hexosaminidase enzyme release and cell viability of RBL-2H3 cells treated with various AGP and DDAG derivatives via calcium Ionophore A23187 stimulation method

Compounds (10 µM)	Inhibition of β-Hexosaminidase Enzyme Release (%)	Viability of RBL-2H3 Cells (%)
AGP	11.1 ± 5.7	81.3
DDAG	15.7 ± 2.1	99.6
SRS21	16.1 ± 9.6	46.2
SRS30	1.6 ± 4.6	57.2
SRS50	9.5 ± 4.8	61.2
SRS77	15.7 ± 1.7	75.1
SRS83	20.7 ± 9.5	109.3
SRS133	53.8 ± 1.9	102.2
SRS143	85.2 ± 3.1	107.2
Quercetin (50 µM)	78.8 ± 8.5	106.3

**Fig. 5:**
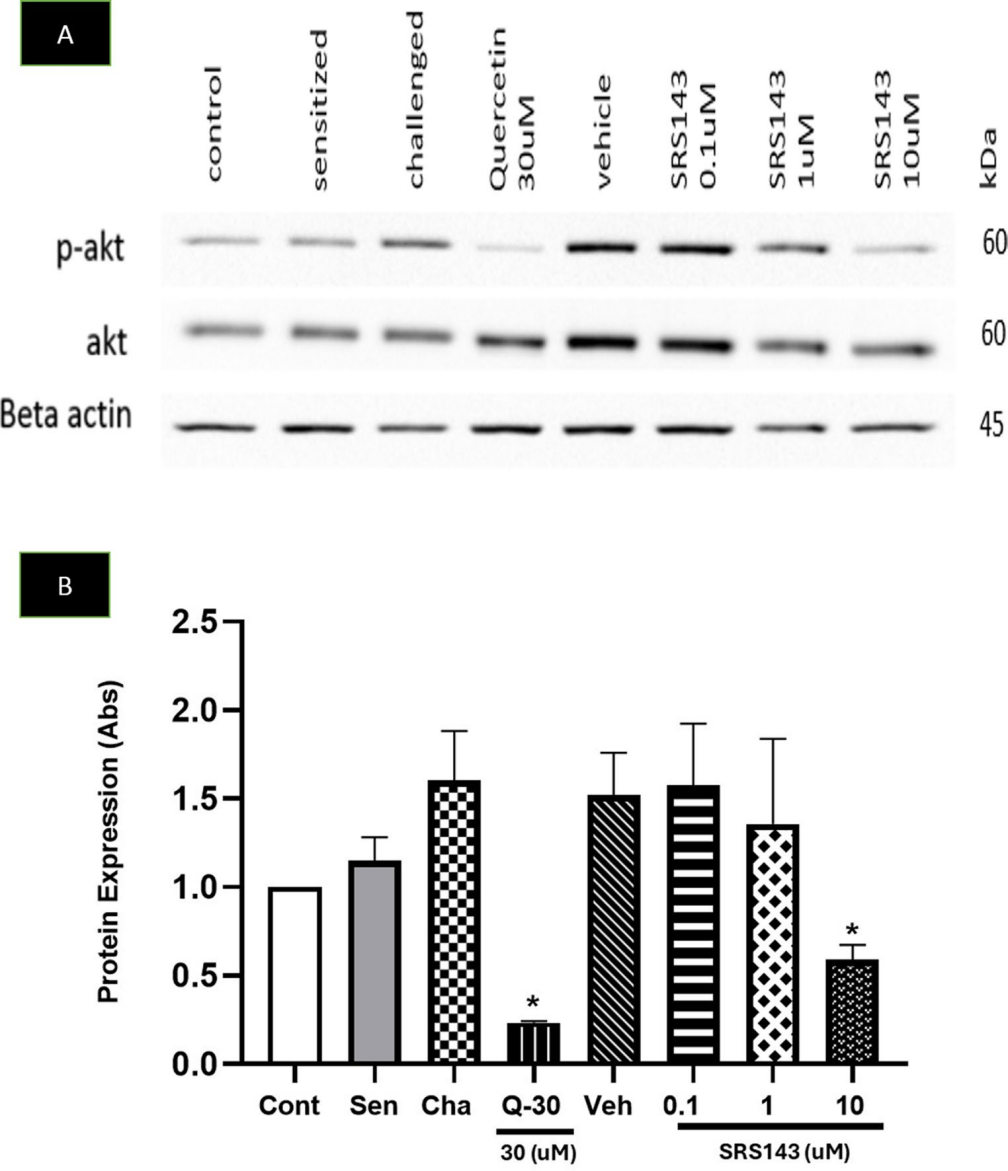
Representative images of Western blot (**A**) and it’s semi-quantitative analysis using image J (**B**) of naïve (Cont) or sensitized (Sen) and IgE-FcεRI activated (Cha) RBL-2H3 cells treated with 0.01% DMSO (Veh), 0.1, 1 and 10 uM of SRS143. SRS143 mediates Akt phosphorylation in activated RBL-2H3 cells whereby the levels of phosphorylated-Akt in IgE-FcεRI mediated activation mast cell RBL-2H3 decrease significantly at the highest dose of 10 μM (n = 3). 30uM of Quercetin (Q-30) served as positive control showed similar inhibition against activated RBL-2H3 cells. Significant difference compared to challenged group. *p < 0.001

In the dose–response efficacy study, SRS143 was found to effectively inhibit histamine release in both calcium ionophore A23187 and Ige-FcεRI receptor stimulation assays at a dose dependent manner without toxicity (Fig. 4). SRS143 had an inhibition of enzyme release of almost 97% at 30 µM having an IC_50_ value of 6.5 µM which is 2 times lower than the IC_50_ value of quercetin overall having a much better activity than quercetin, a flavanoid which has been shown to have antihistamine activity (Fig. 4B). The phosphoinositide 3-kinase signalling pathway is triggered to initiate mast cell degranulation upon binding of allergen on IgE receptor FcεRI. Akt phosphorylation is one of the intermediate signalling process of Phosphoinositide 3-kinase signalling pathway (Sulaiman et al. 2024). Our findings indicate that SRS143 may inhibit mast cell degranulation via antigen activation, this was further supported by the reduction of AKT phosphorylation at a dose dependent manner (Fig. 5). We also attempted to evaluate the toxicity of SRS143 for an incubation period of more than 1 h. The cells were subjected to 1 h, 24 h, and after washing at 24 h incubation. The cytotoxicity study indicated that SRS143 is only toxic if applied at the highest concentration of 100 µM.

### SRS143 inhibits house dust mite-induced asthmatic response

**Fig. 6:**
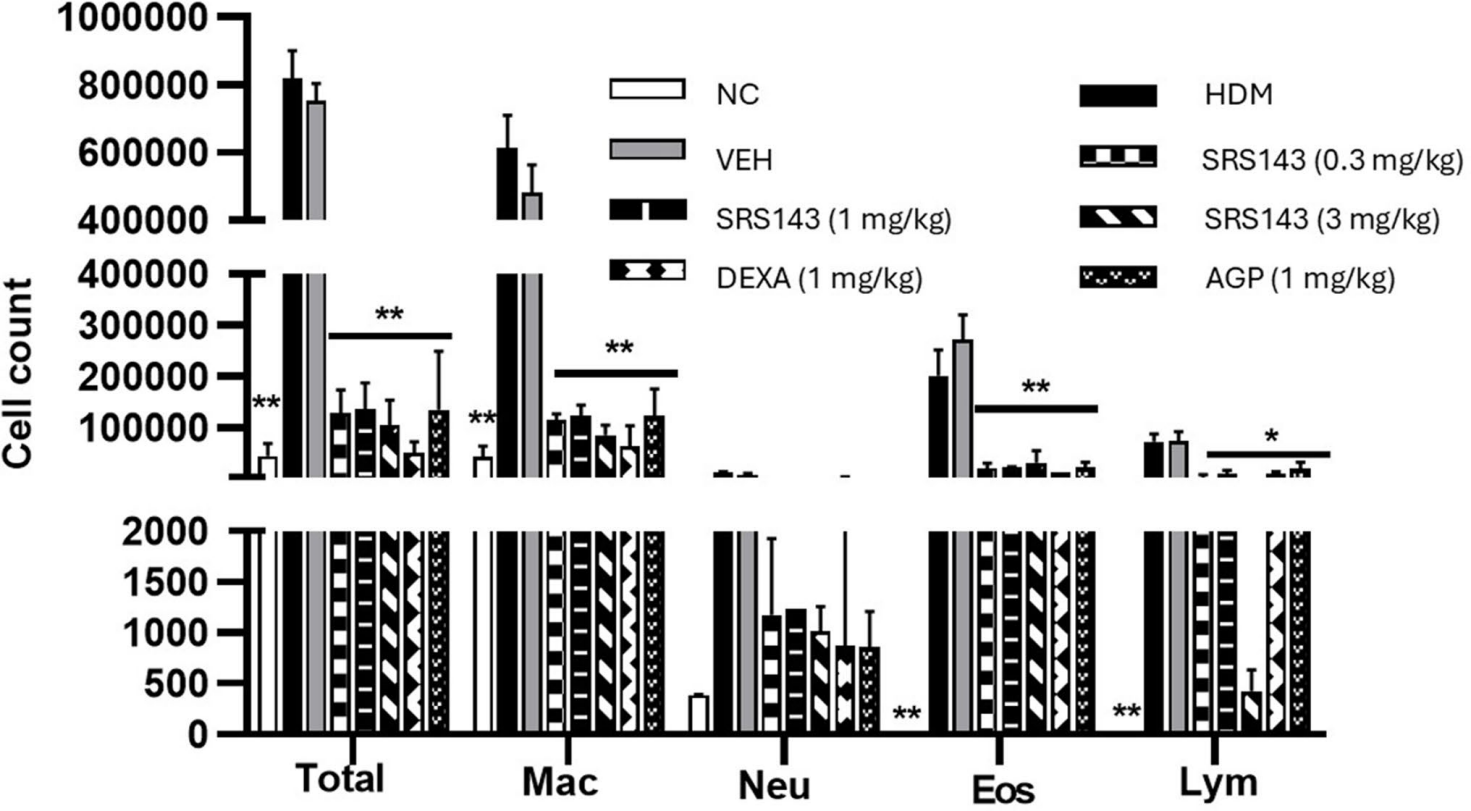
BALF total and differential inflammatory leukocyte counts in SRS143 treated HDM sensitized and challenged mice. Significant increase in total and differential leukocyte counts in HDM sensitisation and challenged or Vehicle Control (6% DMSO). SRS143 dose-dependently reduced macrophage, neutrophil, eosinophil and lymphocytes infiltration in asthmatic airway. Dexamethasone and AGP served as positive control also showed inhibition in airway infiltration of all inflammatory leukocytes. Values shown are the mean ± SD (n = 6). Significantly different from HDM group *p < 0.05, *p < 0.001. Mac—macrophage, Neu—neutrophil, Eos—eosinophil, Lym—lymphocytes

**Fig. 7:**
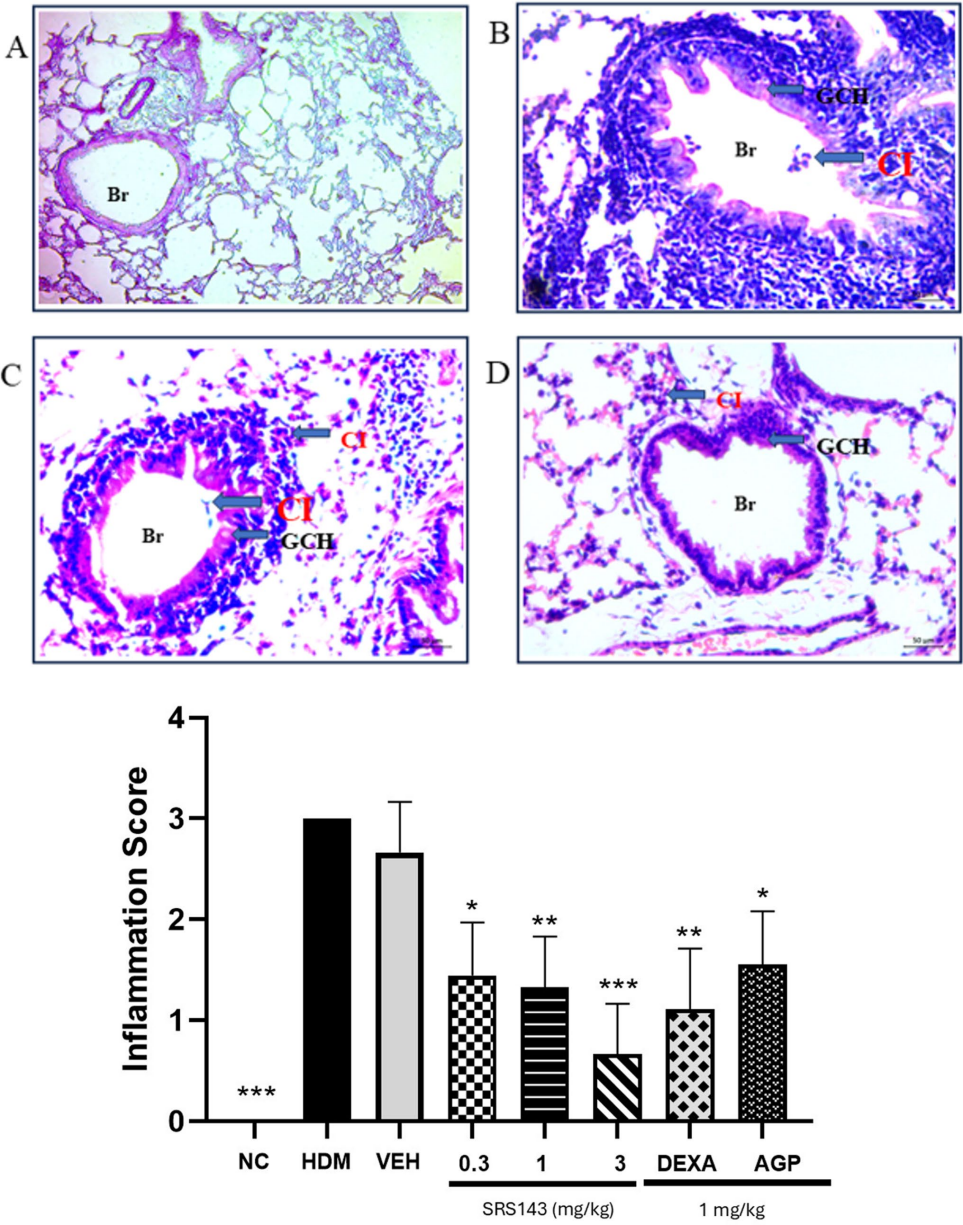
Pictomicrographic representation of lungs from normal control (**A**), Normal control (**B**) HDM induced asthma animals (**C**), vehicle control and (**D**) SRS143 3.0 mg/kg treated animals. Showing hematoxylin and eosin (H&E) staining of inflammatory cells and lung parenchyma. There is evident accumulation of cellular infiltrates in the peribronchial and perivascular regions of HDM exposed mice. An increase in macrophages, lymphocytes, and eosinophils were observed in HDM exposed animals and vehicle group. Br, bronchioles, GCH, globlet cell hyperplasia, CI, cellular infiltration. The treatment reduced peribronchial and perivascular cellular infiltrates level following administration SRS143 3.0 mg/kg. Score interpretation: 0 = no detectable inflammation, 1 = mild inflammation (occasional inflammatory cells in peribronchial space), 2 = moderate inflammation (1- 2 layers of 5–20 inflammatory cells in peribronchial space), 3 = severe inflammation (> 2 layers of more than 20 inflammatory cells in peribronchial space). Values shown are the mean ± SD (n = 6). Values are significantly different from HDM group, *p < 0.05, **p < 0.001, ***p < 0.0001

**Fig. 8:**
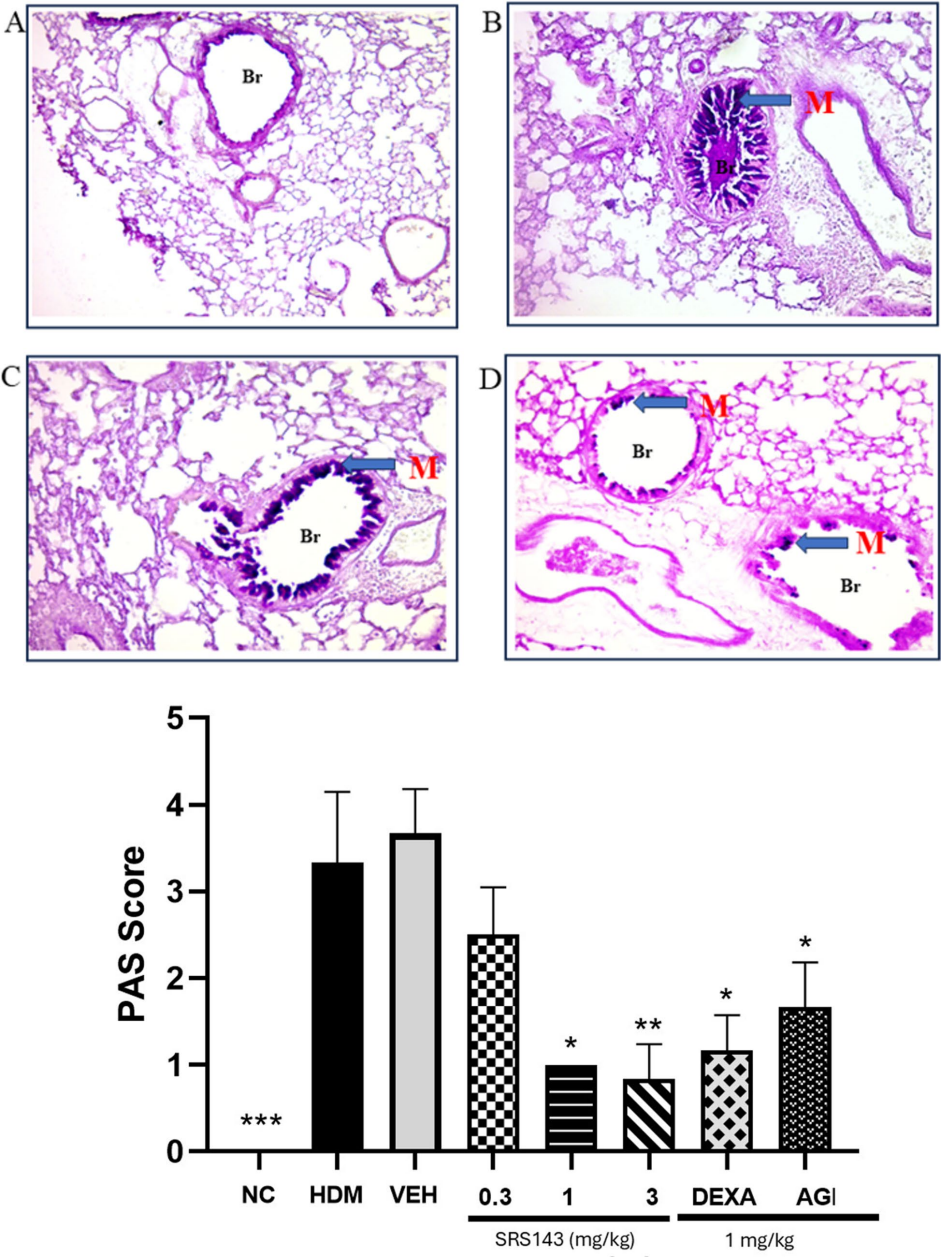
Pictomicrographic representation of lungs from normal control (**A**), HDM induced asthma animals (**B**), vehicle control (**C**) and SRS143 3.0 mg/kg treated animals (**D**). Showing periodic acid Schiff (PAS) stained lung sections. Exposure to HDM (**B**) and (**C**) resulted in mucus accumulation in the airway lumen and induced goblet cell metaplasia. Normal control subjects showed clear lumen and no conspicuous goblet metaplasia. The treatment reduced peribronchial and perivascular cellular infiltrates level. Suppression of airway mucus secretion in SRS143 3.0 mg/kg (**D**) treated HDM sensitized and challenged subjects. Representative pictomicrograph showed decreased bronchial (Br) mucus plugins formation (M) and goblet metaplasia. The mucus and goblet cells (both magenta in colour) stained positive to PAS stain. Score interpretation: 0 = no goblet metaplasia, 1 =  < 25% PAS positive airway goblet cells, 2 = 25%- 49% PAS positive airway goblet cells, 3 = 50%−75% PAS positive airway goblet cells, 4 =  > 75% PAS positive airway goblet cells (Okwuofu et al. 2022). Values shown are the mean ± SD (n = 3). Significantly different from HDM group, *p < 0.05, **p < 0.001

House dust mite (HDM) has been a major source of environmental aeroallergen, a key trigger in the exacerbation of allergic respiratory disease (Jin et al. 2020). HDM has been identified as a risk factor for persistent asthma in human subjects. Compared to one of the most widely used inducible asthma models, the ovalbumin (OVA)-induced asthma model, HDM as allergen of choice to induce airway hyperresponsiveness is more clinically relevant. Similar to OVA-induced asthma models, HDM-sensitized and challenged mice also display an increase in inflammatory cells infiltration. Under cytological examination in HDM-sensitized and challenged mice, almost all inflammatory cells including macrophages, neutrophils, eosinophils, and lymphocytes had significant increase in number of cells in comparison to HDM-sensitized and saline-challenged mice. The overall increase of cell counts for total cells, macrophages, eosinophils, neutrophils, and lymphocytes indicated severe inflammation has taken place in the lung of HDM-challenged animals post-allergen challenge. To assess the effects of SRS143 on the HDM-induced asthmatic response, BAL fluid was collected 24 h after the last HDM or saline challenge. The BAL fluid of lungs of animals treated with SRS143 displayed a significant reduction of total cells, macrophage, neutrophil, eosinophils and lymphocytes infiltration in HDM-induced asthmatic airway (Fig. 6). To further strengthen the observed inflammatory responses BAL fluid samples, the lung tissues were examined for asthma-related histopathological aberrations. The lungs from the mice that were exposed to HDM were severely inflamed. It was evident that HDM induced epithelial cell hypertrophy, peribronchial and perivascular accumulation of inflammatory cells. Furthermore, semi-quantitative analysis of airway inflammatory cells showed increased accumulation of mononuclear leucocytes, eosinophils, and the occasional presence of neutrophils in untreated HDM-induced mice relative to normal control and treated groups. Administration of SRS143 dose-dependently decreased the inflammatory cellular infiltration and recruitment thereby reducing both peribronchial and perivascular inflammation in lung sections. The impact of the treatments on airway inflammation scores was described in Fig. 7. Histopathological analyses of mucus production, accumulation, and goblet metaplasia showed a severe increase in PAS-positive cells among the animals in the HDM group. This variation was significantly attenuated by administration of SRS143. Treatment with SRS143 produced PAS-positive cells score that was between mild to no observable goblet metaplasia (Fig. 8).

**Fig. 9:**
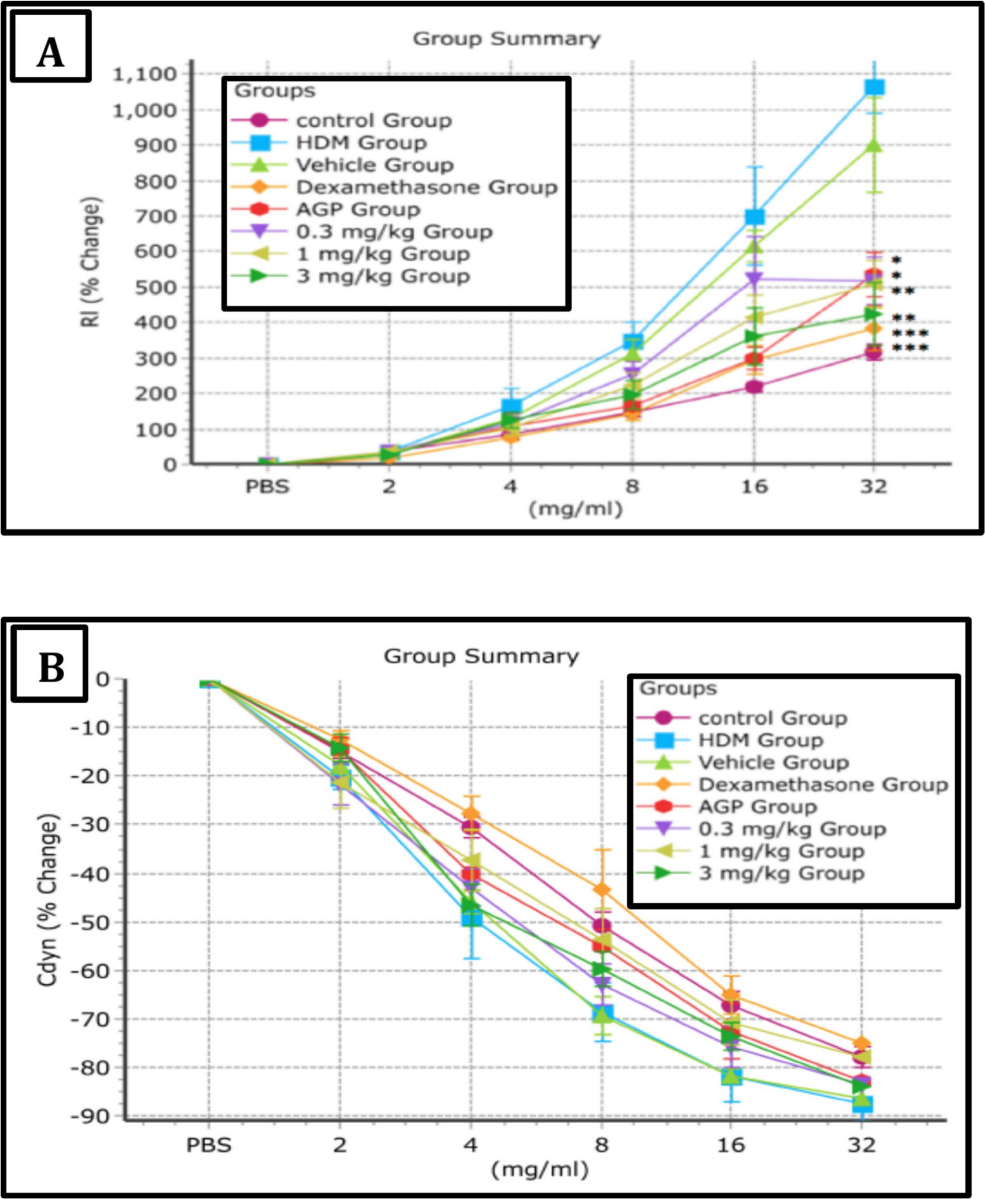
Airway Resistance (**A**) and Dynamic Compliance (**B**) of mechanically ventilated SRS143 treated mice in response to increasing concentration of Methacholine (2.0,4.0,8.0,16.0 and 32.0 mg/ml). There was a remarkable decrease in airway resistance at treatment dose of 1 and 3 mg/kg of SRS143 while dynamic compliance of the lung has no significant changes. Values shown are the mean ± SD (n = 6). Significantly different from HDM group, *p < 0.05, **p < 0.01,***p < 0.001

Airway hyperreactivity (AHR) was analysed using the RC Buxco airway hyper-responsiveness test system. The test showed that HDM sensitization and multiple challenges substantially increased airway resistance (RI) by about 4 folds and slightly decreased dynamic compliance (Cdyn) of the lungs after exposure towards increase dose of methacholine (Fig. 9). Methacholine activates mast cells to release 5-HT, which by acting on 5-HT2A receptors enhances bronchoconstriction and AHR (Wilson and Platts-Mills 2018). The administration of SRS143 resulted in the improvement of RI. The impact of SRS143 treatment on Cdyn was marginal. Change in RI and Cdyn of the treated animals are illustrated in Fig. 9A and B respectively.

## Discussion

This study demonstrated that SRS143 attenuates inflammatory cell infiltration in BAL fluid, suppresses goblet cells hyperplasia, and decreases airway hyperresponsiveness in the lungs of asthmatic mice. It also inhibits Akt, p-Akt, and β-hexosaminidase activity in an in vitro mast cell model. Mast cells and basophils represent the most relevant sources of histamine and β-hexosaminidase in the immune system and are released following the degradation of mast cells. Histamine is a small peptide, which acts as a key mediator in allergic disorders such as allergic rhinitis and asthma. This notorious allergic mediator is stored in cytoplasmic granules along with other amines, proteases, proteoglycans, cytokines/chemokines, angiogenic factors and rapidly released upon triggering with a variety of stimuli (Mendez-Enriquez et al. 2021). Histamine exerts its biological activities by activating four G protein-coupled receptors, namely histamine 1 receptor (H1R), H2R, H3R (expressed mainly in the brain), and the recently identified H4R (Mendez-Enriquez et al. 2021). The H1R and H2R have been reported to be involved mainly in the pathophysiology of allergic diseases such as asthma (Borriello et al. 2017). Through activation of these receptors, the airway undergoes changes such as smooth muscle cell contraction, vasodilatation, increased venular permeability, and mucus hyper-secretion (Yamauchi and Ogasawara 2019). As such, prevention of mast cell activation via inhibiting the cross-linking of antigen-specific IgE bound to high-affinity receptor (FcεRI) on their membranes can be an effective therapeutic strategy in managing allergic asthma (Nguyen et al. 2021; Nagata and Suzuki 2022).

AGP has been shown to display strong anti-inflammatory activity in ovalbumin-induced mouse asthma models (Okwuofu et al. 2022) via NF-kB signaling pathway at the level of inhibitory κB kinase-β activation (Okwuofu et al. 2022). Interestingly, the parent compound AGP did not show any in vitro antihistamine activity in both DNP-IgE and calcium ionophore activated RBL-2h3 cells. This is further supported by a study on HDM induced skin inflammation. Treatment of AGP not only significantly reduce inflammation via NF-kB but it is also capable of restoring the skin barrier functions (Bao et al. 2009). Nevertheless, there were no studies involving AGP in HDM-induced asthma model. Strong inhibition of asthmatic response in HDM animals by water extract of AP had been reported (Lim et al. 2016). However, the inhibition of AP extract was not associated with a direct inhibition of mast cells, but a significant inhibition of NF-kB signaling pathway by preventing IKK phosphorylation, IĸB-α activation, p65 nuclear translocation, and DNA binding activity of p65.

Compounds that inhibit histamine release have been shown to be effective in preventing allergic asthma in a mouse model (Bayazid and Jang 2021). SRS143 is a novel AGP analogue with potent inhibitory activity in an in vitro mast cell degranulation. Our results strongly indicated that this analogue is capable of direct inhibiting mast cell degranulation. The inhibitory activity of SRS143 against β-hexosaminidase release from mast cell, may be due to the benzylidene and acetyl moiety attached to position 19 and 14 of the parent compound respectively. This study shows a strong inhibition of HDM asthma model, whereby SRS143 inhibited inflammatory cells recruitment and infiltration, goblet cells hyperplasia, and airway hyperresponsiveness. A similar result was obtained in an OVA model of asthma, H4R antagonist reduced inflammatory activity when dosed prophylactically in an acute mouse model and demonstrated a reduction in Th2 cytokine production (Sang et al. 2022; Dunford et al. 2006; Cowden et al. 2010).

Our previous hallmark study of another AGP analogue, SRS27 in same ovalbumin induced mouse model reported that SRS27 had a similar mechanism of action like AGP by targeting NF-kB signaling pathway in TNF-alpha activated A549 cells. Treatment of SRS27 only resulted in significant reduction of macrophages and eosinophils in BAL fluid from the lungs of OVA induced and challenged animals (Matsuda et al. 1994). Besides, ovalbumin specific IgE and total IgE were found down regulated in the OVA induced and challenged animals, an indication of SRS27 indirect mediation on mast cell activity.

**Fig. 10:**
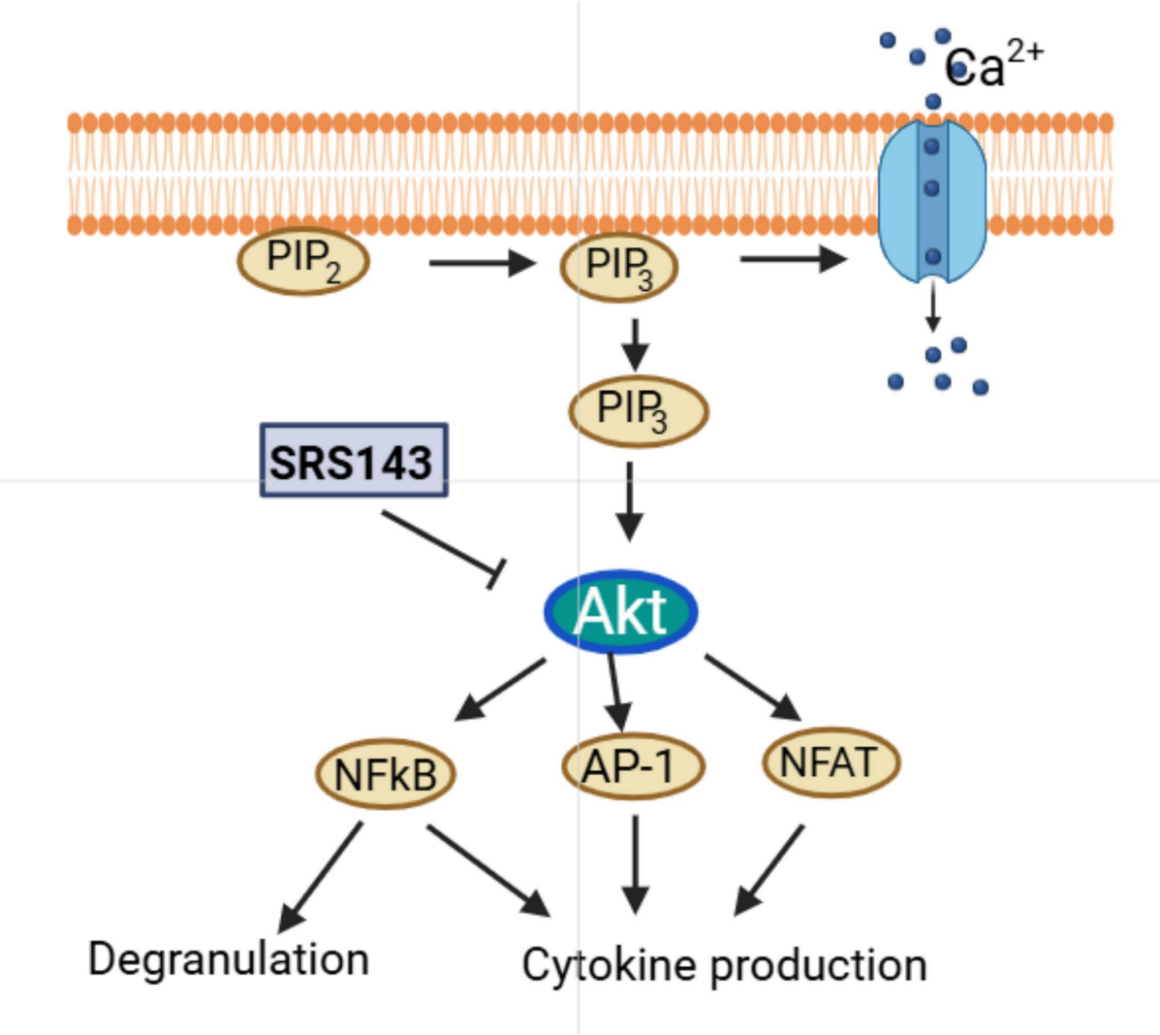
A proposed mechanism of action of SRS143 for the prevention of asthma via inhibiting mast cell degranulation and Akt protein

SRS143 an analogue of AGP has demonstrated a remarkable potency in inhibiting histamine release, suggesting a potentially distinct mechanism of action compared to AGP. While the exact mechanism remains unconfirmed, we hypothesized that Akt phosphorylation is linked to the anti-histamine activity of SRS143 in mast cells. By targeting the Akt pathway, SRS143 inhibits Akt phosphorylation P13K, a crucial step in downstream signaling pathway (Fig. 10). This inhibition prevents NF-κB activation, a transcription factor regulating pro-inflammatory cytokines and mediators (Fig. 10). Consequently, SRS143 suppresses airway inflammation and goblet cells hyperplasia, and airway hyperresponsiveness, thereby alleviating key pathological features of asthma.

## Conclusion

SRS143 demonstrated strong anti-asthmatic effects in both in vitro and in vivo models by effectively suppressing histamine release through the inhibition of mast cell degranulation and activation. This mechanism mitigated allergy-induced airway hyperresponsiveness in an HDM-induced asthma model. Moreover, SRS143 has been shown to target and inhibit the activity of Akt protein in the PI3K/Akt signaling pathway, making SRS143 a potential candidate for asthma therapy.

## Supplementary Information

Below is the link to the electronic supplementary material.

Supplementary file1 (DOCX 466 KB)

## Data Availability

All source data for this work (or generated in this study) are available upon reasonable request.

## References

[CR1] Akar-Ghibril N et al (2020) Allergic endotypes and phenotypes of asthma. J Allergy Clin Immunol Pract 8(2):429–440 32037107 10.1016/j.jaip.2019.11.008 PMC7569362

[CR2] Asher MI et al (2020a) Trends in worldwide asthma prevalence. Eur Respir J 56(6):1–14 10.1183/13993003.02094-2020 32972987

[CR3] Asher MI et al (2020b) Trends in worldwide asthma prevalence. Eur Respir J. 10.1183/13993003.02094-2020 32972987 10.1183/13993003.02094-2020

[CR4] Banafea GH et al (2022) The role of human mast cells in allergy and asthma. Bioengineered 13(3):7049–7064 35266441 10.1080/21655979.2022.2044278 PMC9208518

[CR5] Bao Z et al (2009) A novel antiinflammatory role for andrographolide in asthma via inhibition of the nuclear factor-kappaB pathway. Am J Respir Crit Care Med 179(8):657–665 19201922 10.1164/rccm.200809-1516OC

[CR6] Bax HJ, Keeble AH and Gould HJ (2012) Cytokinergic IgE action in mast cell activation. Front Immun 3:229. 10.3389/fimmu.2012.00229 10.3389/fimmu.2012.00229 PMC3412263 22888332

[CR7] Bayazid AB, Jang YA (2021) The role of Andrographolide on skin inflammations and modulation of skin barrier functions in human keratinocyte. Biotechnol Bioprocess Eng 26(5):804–813

[CR8] Borriello F, Iannone R, Marone G (2017) Histamine release from mast cells and basophils. Handb Exp Pharmacol 241:121–139 28332048 10.1007/164_2017_18

[CR9] Chandrasekaran CV et al (2011) In vitro modulation of LPS/calcimycin induced inflammatory and allergic mediators by pure compounds of Andrographis paniculata (king of bitters) extract. Int Immunopharmacol 11(1):79–84 21034865 10.1016/j.intimp.2010.10.009

[CR10] Cowden JM et al (2010) Histamine H4 receptor antagonism diminishes existing airway inflammation and dysfunction via modulation of Th2 cytokines. Respir Res 11(1):86 20573261 10.1186/1465-9921-11-86 PMC2914735

[CR11] Dharmage SC, Perret JL, Custovic A (2019) Epidemiology of asthma in children and adults. Front Pediatr 7:246 31275909 10.3389/fped.2019.00246 PMC6591438

[CR12] Dinglasan JL et al (2022) Asthma prevalence and the relationship between level of knowledge and quality of life among asthmatic school children in Malaysia. Saudi Med J 43(1):113–116 35022293 10.15537/smj.2022.43.1.20210211 PMC9280567

[CR13] Dunford PJ et al (2006) The histamine H4 receptor mediates allergic airway inflammation by regulating the activation of CD4+ T cells. J Immunol 176(11):7062–7070 16709868 10.4049/jimmunol.176.11.7062

[CR14] García-Marcos L et al (2022) The burden of asthma, hay fever and eczema in children in 25 countries: GAN phase i study. Eur Respir J. 10.1183/13993003.02866-2021 35144987 10.1183/13993003.02866-2021 PMC9474895

[CR15] García-Marcos L et al (2023) Asthma management and control in children, adolescents, and adults in 25 countries: a Global Asthma Network Phase I cross-sectional study. Lancet Glob Health 11(2):e218–e228 36669806 10.1016/S2214-109X(22)00506-X PMC9885426

[CR16] Gold LS et al (2014) Level of asthma control and health care utilization in Asia-Pacific countries. Respir Med 108(2):271–277 24406243 10.1016/j.rmed.2013.12.004

[CR17] Gupta S et al (2020) Andrographolide attenuates complete freund’s adjuvant induced arthritis via suppression of inflammatory mediators and pro-inflammatory cytokines. J Ethnopharmacol 261:113022 32569719 10.1016/j.jep.2020.113022

[CR18] Hu X et al (2015) Evaluation of the anaphylactoid potential of Andrographis paniculata extracts using the popliteal lymph node assay and P815 cell degranulation in vitro. J Transl Med 13(1):121 25889593 10.1186/s12967-015-0478-0 PMC4409753

[CR19] Jiaqi L et al (2023) Andrographolide promoted ferroptosis to repress the development of non-small cell lung cancer through activation of the mitochondrial dysfunction. Phytomedicine 109:154601 36610134 10.1016/j.phymed.2022.154601

[CR20] Jin JR et al (2020) PI3Kγ regulatory protein p84 determines mast cell sensitivity to Ras inhibition-moving towards cell specific PI3K targeting? Front Immunol 11:585070 33193405 10.3389/fimmu.2020.585070 PMC7655736

[CR21] Karpakavalli M et al (2010) Anti-histamine effect of hydroalcoholic extract of Andrographis paniculata leaf (Burm. F). Der Chemica Sinica

[CR22] Lim JC et al (2012) Andrographolide and its analogues: versatile bioactive molecules for combating inflammation and cancer. Clin Exp Pharmacol Physiol 39(3):300–310 22017767 10.1111/j.1440-1681.2011.05633.x

[CR23] Lim J-W et al (2016) A semisynthetic diterpenoid lactone inhibits NF-κB signalling to ameliorate inflammation and airway hyperresponsiveness in a mouse asthma model. Toxicol Appl Pharmacol 302:10–22 27089844 10.1016/j.taap.2016.04.004

[CR24] Lin KH et al (2020) Andrographolide mitigates cardiac apoptosis to provide cardio-protection in high-fat-diet-induced obese mice. Environ Toxicol 35(6):707–713 32023008 10.1002/tox.22906

[CR25] Matsuda T et al (1994) Cell differentiation-inducing diterpenes from Andrographis paniculata Nees. Chem Pharm Bull (Tokyo) 42(6):1216–1225 8069972 10.1248/cpb.42.1216

[CR26] Mendez-Enriquez E et al (2021) Mast cell-derived serotonin enhances methacholine-induced airway hyperresponsiveness in house dust mite-induced experimental asthma. Allergy 76(7):2057–2069 33486786 10.1111/all.14748

[CR27] Nagata Y, Suzuki R (2022) FcεRI: a master regulator of mast cell functions. Cells. 10.3390/cells11040622 35203273 10.3390/cells11040622 PMC8870323

[CR28] Nguyen SMT et al (2021) Mechanisms governing anaphylaxis: inflammatory cells, mediators, endothelial gap junctions and beyond. Int J Mol Sci. 10.3390/ijms22157785 34360549 10.3390/ijms22157785 PMC8346007

[CR29] Okwuofu EO et al (2022) Molecular and immunomodulatory actions of new antiasthmatic agents: exploring the diversity of biologics in Th2 endotype asthma. Pharmacol Res 181:106280 35661709 10.1016/j.phrs.2022.106280

[CR30] Pejler G (2019) The emerging role of mast cell proteases in asthma. Eur Respir J. 10.1183/13993003.00685-2019 31371445 10.1183/13993003.00685-2019

[CR31] Phetruen T, van Dam B, Chanarat S (2023) Andrographolide induces ROS-mediated cytotoxicity, lipid peroxidation, and compromised cell integrity in Saccharomyces cerevisiae. Antioxidants. 10.3390/antiox12091765 37760068 10.3390/antiox12091765 PMC10525756

[CR32] Poetker DM, Reh DD (2010) A comprehensive review of the adverse effects of systemic corticosteroids. Otolaryngol Clin North Am 43(4):753–768 20599080 10.1016/j.otc.2010.04.003

[CR33] Sang X et al (2022) Screening of bioactive fraction of Radix Paeoniae Alba and enhancing anti-allergic asthma by stir-frying through regulating PI3K/AKT signaling pathway. Front Pharmacol 13:863403 35431951 10.3389/fphar.2022.863403 PMC9009445

[CR34] Sulaiman I et al (2024) An Andrographis paniculata Burm. Nees extract standardized for three main Andrographolides prevents house dust mite-induced airway inflammation, remodeling, and hyperreactivity by regulating Th1/Th2 gene expression in mice. J Ethnopharmacol 319:117082–117093 37652197 10.1016/j.jep.2023.117082

[CR35] Sze E, Bhalla A, Nair P (2020) Mechanisms and therapeutic strategies for non-T2 asthma. Allergy 75(2):311–325 31309578 10.1111/all.13985

[CR36] Wilson JM, Platts-Mills TAE (2018) Home environmental interventions for house dust mite. J Allergy Clin Immunol Pract 6(1):1–7 29310755 10.1016/j.jaip.2017.10.003 PMC6474366

[CR37] Yamauchi K, Ogasawara M (2019) The role of histamine in the pathophysiology of asthma and the clinical efficacy of antihistamines in asthma therapy. Int J Mol Sci. 10.3390/ijms20071733 30965592 10.3390/ijms20071733 PMC6480561

[CR38] Yasir M, Goyal A, Sonthalia S (2024) Corticosteroid adverse effects, in StatPearls. StatPearls Publishing Copyright © 2024, StatPearls Publishing LLC.: Treasure Island (FL) ineligible companies. Disclosure: Amandeep Goyal declares no relevant financial relationships with ineligible companies. Disclosure: Sidharth Sonthalia declares no relevant financial relationships with ineligible companies

