# Supplementary figures and images for: SRS143 a semi-synthetic analogue of andrographolide against house dust mite induced allergic asthma
Source: Naunyn Schmiedebergs Arch Pharmacol. 2025 Oct 25;399(4):4957–71. doi: 10.1007/s00210-025-04589-8 (PMC13046583; doi:10.1007/s00210-025-04589-8)

Western Blot

N=1


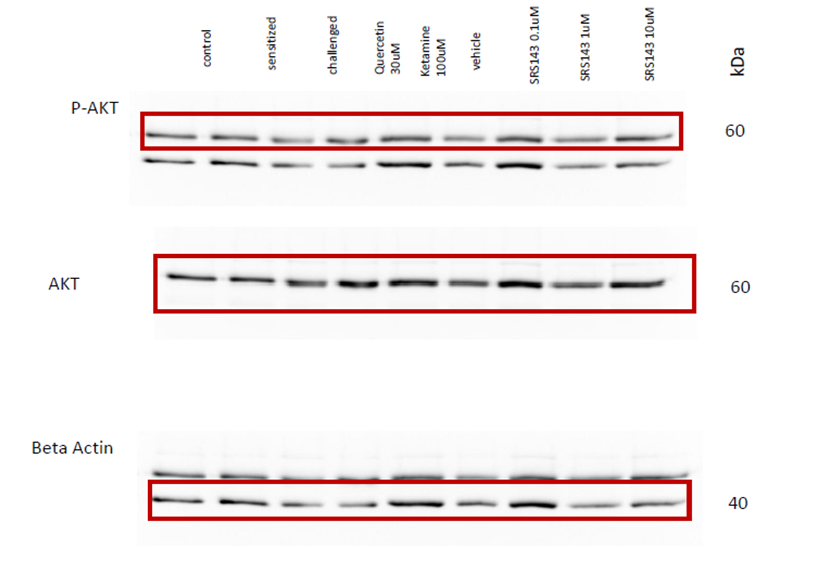


N=2


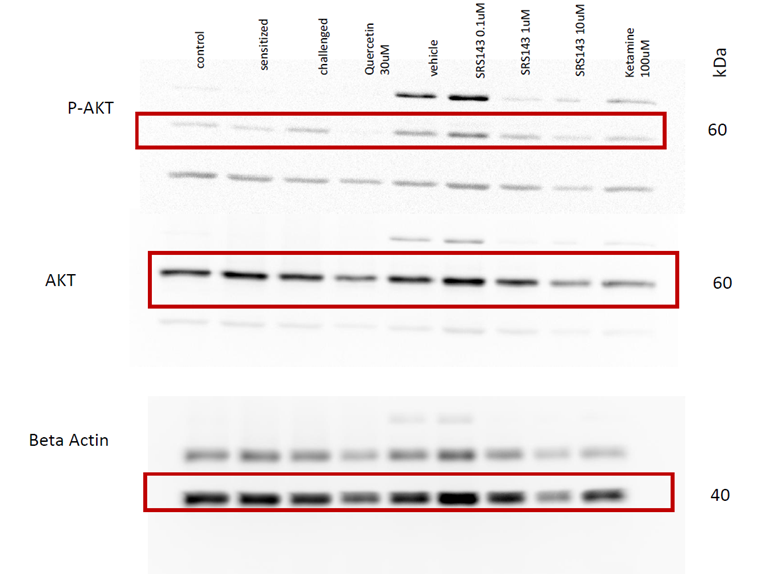


N=3


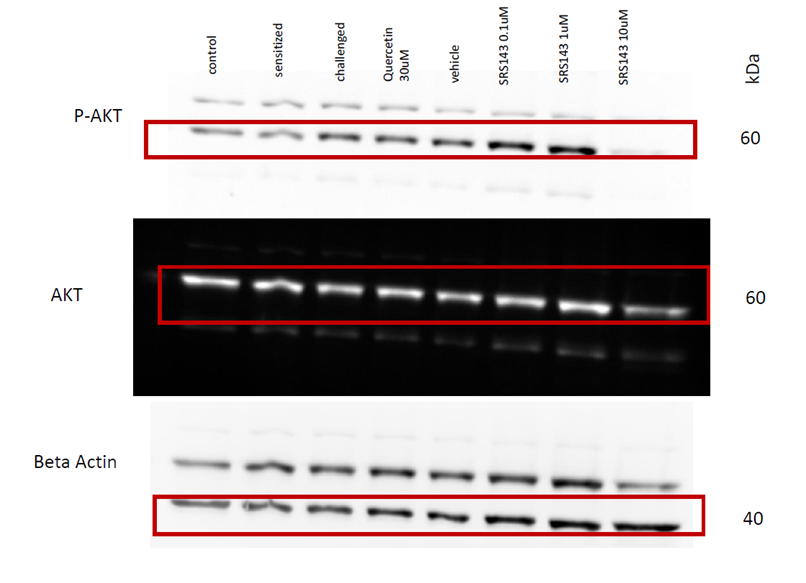

Supplement: Supplementary file 1 — Supplementary file1 (DOCX 466 KB) [file 210_2025_4589_MOESM1_ESM.docx]
